# Steroid hormone receptors ERα and PR characterised by immunohistochemistry in the mare adrenal gland

**DOI:** 10.1186/1751-0147-51-31

**Published:** 2009-07-22

**Authors:** Ylva Hedberg Alm, Sayamon Sukjumlong, Hans Kindahl, Anne-Marie Dalin

**Affiliations:** 1 University Animal Hospital, Swedish University of Agricultural Sciences, P.O. Box 7040, SE-750 07 Uppsala, Sweden; 2 Division of Reproduction, Department of Clinical Sciences, Swedish University of Agricultural Sciences, P.O. Box 7054, SE-750 07 Uppsala, Sweden; 3 Department of Anatomy, Faculty of Veterinary Science, Chulalongkorn University, Bangkok, Thailand

## Abstract

**Background:**

Sex steroid hormone receptors have been identified in the adrenal gland of rat, sheep and rhesus monkey, indicating a direct effect of sex steroids on adrenal gland function.

**Methods:**

In the present study, immunohistochemistry using two different mouse monoclonal antibodies was employed to determine the presence of oestrogen receptor alpha (ERalpha) and progesterone receptor (PR) in the mare adrenal gland. Adrenal glands from intact (n = 5) and ovariectomised (OVX) (n = 5) mares, as well as uterine tissue (n = 9), were collected after euthanasia. Three of the OVX mares were treated with a single intramuscular injection of oestradiol benzoate (2.5 mg) 18 – 22 hours prior to euthanasia and tissue collection (OVX+Oe). Uterine tissue was used as a positive control and showed positive staining for both ERalpha and PR.

**Results:**

ERalpha staining was detected in the adrenal zona glomerulosa, fasciculata and reticularis of all mare groups. Ovariectomy increased cortical ERalpha staining intensity. In OVX mares and one intact mare, positive ERalpha staining was also detected in adrenal medullary cells. PR staining of weak intensity was present in a low proportion of cells in the zona fasciculata and reticularis of all mare groups. Weak PR staining was also found in a high proportion of adrenal medullary cells. In contrast to staining in the adrenal cortex, which was always located within the cell nuclei, medullary staining for both ERalpha and PR was observed only in the cell cytoplasm.

**Conclusion:**

The present results show the presence of ERalpha in the adrenal cortex, indicating oestradiol may have a direct effect on mare adrenal function. However, further studies are needed to confirm the presence of PR as staining in the present study was only weak and/or minor. Also, any possible effect of oestradiol treatment on the levels of steroid receptors cannot be determined by the present study, as treatment time was of a too short duration.

## Background

Activation of the hypothalamic-pituitary-adrenal (HPA) axis, with the release of ACTH and cortisol, as occurs during stress, often has an inhibitory effect on the reproductive system [1-3]. The interaction between the HPA axis and the hypothalamic-pituitary-gonadal (HPG) axis may act in both ways, with reproductive hormones also influencing adrenal function. The presence of adrenal sex steroid hormone receptors in the adrenal gland may give an answer to whether sex steroid hormones can act directly on the adrenal gland.

Using a LBA, ERs were found in the adrenal gland of the rat [4]. Further, using IHC, ERs were found to be localised within cell nuclei of the adrenal cortex of both rhesus monkey [5] and sheep [6]. In the study of Van Lier et al. [6], results suggested that both known subtypes of ER, ERα and ERβ, were present in the sheep adrenal gland.

In addition to ER, other adrenal sex steroid hormone receptors have been demonstrated in some species, such as the androgen receptor (AR) in the rat [7], rhesus monkey [5] and human [8]. Using a solution hybridisation assay, progesterone receptor mRNA (PR mRNA) was detected in the sheep adrenal [6]. Likewise, PR staining has been observed in adrenal capsular cells in OVX rats [9]. However, in another study, although progesterone binding was detected in adrenocortical nuclei of guinea pig, none was seen in rat, dog, pig and chinchilla [10]. Similarly, using IHC, no adrenal PR staining was found in rhesus monkey [5].

The study of adrenal sex hormone receptors are of interest since their presence or absence indicate whether or not the ovarian hormone fluctuations occurring during the oestrous cycle could have a direct effect on adrenal gland function. To our knowledge, sex steroid receptors in the equine adrenal gland have not yet been investigated. In a previous study, we were not able to detect any effect of endogenous oestradiol on the quantity of adrenal steroid hormones produced when mares (intact in oestrus and after ovariectomy) were treated with a synthetic ACTH (tetracosactide) [11]. However, the basal cortisol pattern differed between mare groups (intact and ovariectomised), suggesting oestradiol may affect basal adrenal function. The aim of the present study was to investigate the presence of ERα and PR in the mare adrenal gland, using IHC.

## Methods

This preliminary study was part of a much larger study investigating adrenal steroid hormone production in mares [11,12]. All of the procedures of this larger study were approved by the Ethical Committee for Experimental Studies with Animals. The animals euthanized in the present study had either been used in the larger study or were mares used in the teaching of veterinary students and were destined for euthanasia regardless. Permission was granted for the collection of organs from all mares used in the present study.

### Experimental animals

Ten healthy mares, with an age span of 3–20 years and weighing between 400–600 kg were used in the study (age was unknown for one mare). Breeds represented were Standardbred trotter (n = 7), New Forest (n = 1), Swedish Warmblood (n = 1) and Thoroughbred (n = 1). Five of the mares were ovariectomised at least six months prior to euthanasia and sample collection. Three of these mares were treated with 0.5 ml of oestradiol benzoate (5 mg/ml) i.m. 18–22 hours before euthanasia (OVX+Oe), with the remaining two mares left untreated (OVX). This treatment period is shorter than the time required for up-regulation of protein levels in other species, but was chosen because of the rapid effect oestradiol is known to have on oestrous behaviour in the mare. The other five mares were intact. Oestrous cycle phase of the intact mares was not known, but blood samples for oestradiol and progesterone analyses were collected prior to euthanasia from all ten mares. Samples were immediately centrifuged and the plasma stored at -18°C until assay. For a summary of the experimental animals used, please see Table 1. All of the OVX mares and two of the intact mares were euthanised with an intravenous injection of Somulose [50 ml; Quinalbarbitone Sodium (400 mg/ml) and Cinchocainehydrochloride (25 mg/ml), Arnolds Veterinary Products Ltd, Harlescott, Shropshire, UK] after sedation with acepromazine [3 ml; Plegicil^®^vet. (10 mg/ml), Pharmaxim Sweden AB, Helsingborg, Sweden], at the Department of Clinical Sciences. One intact mare was euthanised at a slaughter house with a bullet shot and subsequent debleeding. Finally, adrenal glands from two intact mares were collected after anaesthesia [induced with detomidine (Domosedan vet. (10 mg/ml), Orion Pharma Animal Health, Sollentuna, Sweden) and ketamine (Ketaminol^®^vet. (100 mg/ml), Intervet AB, Stockholm, Sweden) and maintained using halothane inhalation] and euthanasia using intravenous injection of pentobarbital sodium (Avlivningsvätska (100 mg/ml), Apoteket AB, Sweden).

**Table 1:** Summary of experimental mares.

ID	Reproductive status	Additional treatment	Plasma progesterone (nmol/l)	Plasma oestradiol (pmol/l)	Evaluation of ovaries
526	Intact -oestrus	None	0.5	16.0	3.5 cm follicle
478	Intact - metoestrus	None	2.7	9.0	Cavitated CL; follicles ≤ 1.5 cm
453	Intact -early dioestrus	None	12.0	7.0	CH
464	Intact -early dioestrus	None	18.9	15.0	CH
502	Intact - dioestrus	None	24.6	13.0	CL; 2.5 cm follicle
740	OVX	None	< 0.1	2.0	---
744	OVX	None	< 0.1	6.0	---
741	OVX+Oe	Oestradiol* 20 h before euthanasia	< 0.1	43.0	---
742	OVX+Oe	Oestradiol* 22 h before euthanasia	< 0.1	35.0	---
743	OVX+Oe	Oestradiol* 18 h before euthanasia	< 0.1	59.0	---

* Oestradiol benzoate (2.5 mg i.m.); OVX: ovariectomised; OVX+Oe: ovariectomised with oestradiol benzoate treatment; CH: corpus haemorrhagicum; CL: corpus luteum.

### Tissue sample collection and fixation

Adrenal glands were collected immediately after euthanasia in all mares and weighed. However, time from collection to fixation of the tissue was from 20 minutes up to one hour, since collection was sometimes difficult due to the deep location of the adrenals. From the intact mares, both ovaries (all mares; n = 5) and uteri (all mares except mare 464; n = 4) were also collected. From OVX mares, uteri were collected (n = 5). All of the tissue samples were fixed in 10% formaldehyde for up to two days and thereafter embedded in paraffin. IHC was performed on adrenal and uterine tissues only, whereas the ovaries were macroscopically examined to aid the determination of cyclic status.

### Hormone analyses

The hormone analyses were performed at the Department of Biomedical Sciences and Veterinary Public Health, Swedish University of Agricultural Sciences, Uppsala, Sweden.

### Progesterone

The concentration of progesterone in peripheral blood plasma was determined using a solid-phase radioimmunoassay (Coat-a-Count Progesterone, Diagnostic Products Corporation, Los Angeles, USA). The kit was used according to the manufacturer's instructions. The relative cross-reactions of the antibody were 0.9% with corticosterone and 0.1% with testosterone. The inter- and intra-assay coefficients of variation for progesterone were as follows: 16.1% and 4.3% at 3.5 nmol/l; 7.3% and 8.5% at 22.5 nmol/l; 23.3% and 6.4% at 54.8 nmol/l. The minimal assay sensitivity of progesterone was 0.15 nmol/l.

### 17-β-Oestradiol

Concentrations of oestradiol were determined by radioimmunoassay using a DPC kit (Diagnostic Product Co., Los Angeles, CA, USA), as reported for use in bovine plasma [13]. The method has previously been validated [14]. All samples were run in duplicates. The inter- and intra-assay coefficients of variation were as follows: 20.0% and 42.5% at 3.2 pmol/l; 7.7% and 5.0% at 46.5 pmol/l; and 12.0% and 6.2% at 123.2 pmol/l. The minimal detectable concentration of oestradiol was 2.1 pmol/l.

### Immunohistochemical procedures

The immunohistochemical procedure has been described previously by Sukjumlong et al. [15]. In brief, the antigen retrieval was performed by heating the sample in 0.01 M citric buffer (pH 6.0) 2 × 5 min in a microwave at 750 watt. Endogenous peroxidase acitivty was blocked with 3% hydrogen peroxide in methanol for 10 minutes. A standard avidin-biotin immunoperoxidase technique (Vectastain^® ^ABC kit, Vector Laboratories Inc., Burlingame, CA, USA) was applied to detect the steroid receptors (ERα and PR). The primary antibodies used were two different mouse monoclonal antibodies to ERα (ERα, C311-sc787, Santa Cruz Biotechnology Inc., Santa Cruz, CA, USA) and PR (PR-2C5, Zymed Laboratories Inc., South San Francisco CA, USA) at the dilution of 1:50 and 1:200, respectively. The incubation time for the primary antibody was 1.5 h at room temperature. A negative control was obtained by replacing the primary antibody with non-immune serum (IgG_2a_) at the same concentration as the primary antibody. The secondary antibody used was a biotinylated horse anti-mouse IgG (Vectastain ABC kit, Vector laboratories Inc., Burlingame, CA, USA) in a dilution of 1:200 for 30 min. In the final step, 3,3'-diaminobenzidine (DAB, Dakopatts AB, Älvsjö, Sweden), a chromogen, was added to visualise the bound enzyme (brown colour) for 3 min, and all uterine sections were counterstained with Mayer's hematoxylin for about 10 seconds. Selected sections were photographed with a Nikon microphot-FXA photomicroscope. Uterine tissue of a mare at oestrus, known to express steroid receptors (ERα and PR) was used as the positive control.

None of the adrenal sections, except for the negative controls, were counterstained since this may in fact have concealed the positive brown nuclei. However, when counterstaining was not performed on the negative sections, it was impossible to identify the cells in these sections. However, for the uterine tissues, the negative and positive cells were clearly seen and positive cells were better comparable after counterstaining with hematoxylin.

### Classification of positively stained cells

The evaluation of ERα and PR positive cells was carried out by the same person (Sayamon Sukjumlong) who was unaware of the identity of the mares. The classification was based on a manual visual evaluation of the sections without the use of any computer software programme.

### Uterus

In uterine tissue, four different compartments were evaluated: the surface epithelium, the glandular epithelium and the connective tissue stroma of the endometrium as well as the myometrium. Staining intensity for uterine tissue was classified as negative (-), weak (+), moderate (++) or strong (+++). The proportion of stained cells in the different uterine compartments were graded as low (<30%), moderate (30–60%), high (>60–90%) or almost all cells (>90%) positive.

### Adrenal gland

In the adrenal glands, the evaluation was performed in both the adrenal cortex and adrenal medulla. In the adrenal cortex, three different zones were examined: zona glomerulosa, zona fasciculata and zona reticularis. No positive staining was found in the adrenal capsule, which is mainly composed of connective tissue and was therefore not included. Due to the relatively weaker staining intensity observed in adrenal tissue, classification as in uterine tissue was not possible. Staining intensity in adrenal tissue was classified as weak or moderate. However, these classifications were not defined as for uterine tissue (the staining intensity was always weaker in adrenal tissue). The proportion of positively stained cells in the different adrenal zones was graded as minor (<50%) or major (>50%).

## Results

### Hormone concentrations and ovary evaluation (intact mares)

The results of the oestradiol and progesterone concentrations and ovarian findings are summarised in Table 1. Three of the intact mares (453, 464 and 502) were judged to be in dioestrus and one mare, in oestrus (526). Progesterone levels in mare 478 were low (< 3 nmol/l) at the time of euthanasia, but the presence of a cavitated corpus luteum indicated recent ovulation, and therefore the mare was determined to be in metoestrus. All OVX and OVX+Oe mares had plasma progesterone concentrations below the minimal detectable level of 0.15 nmol/l. The two untreated OVX mares (740 and 744) had oestradiol levels of 2 pmol/l and 6 pmol/l, respectively. Plasma oestradiol levels were = 35 pmol/l in the three OVX+Oe mares (741, 742 and 743).

### Adrenal tissue

Both adrenal glands were obtained from all but two mares (mares 464 and 740), in which only one adrenal gland, for technical reasons, could be collected. Mean adrenal weight was 17.2 g (SD ± 6.0) (n = 14). Four adrenal glands (from mares 453, 502 and 740) were for technical reasons not weighed.

### Immunohistochemistry – oestrogen receptor alpha (ERα)

#### Uterine tissue

**Figure 1:**
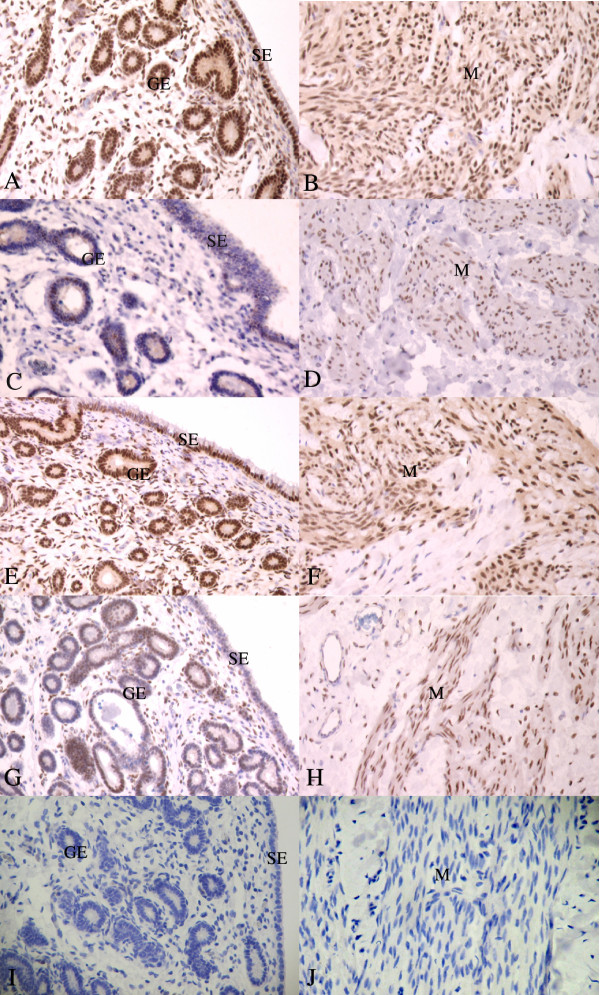
**Immunostaining for ERα and PR in uterine tissue**. A and B: ERα in mare 526 (intact in oestrus); C and D: ERα in mare 478 (intact in metoestrus); E and F: PR in mare 526 (intact in oestrus); G and H: PR in mare 478 (intact in metoestrus); I and J: negative control. SE = surface epithelium; GE = glandular epithelium; M = myometrium. Magnification 200×.

In uterine tissue, positive ERα staining was observed in cell nuclei of all compartments of the endometrium (surface epithelium, glandular epithelium and connective tissue stroma) and myometrium (see Table 2 and Fig. 1). For all mares, the highest proportion of ERα staining was, in general, found in the glandular epithelium and the myometrium. For intact mares, the mare in oestrus (mare 526) showed the strongest staining intensity and the highest proportion of stained cell nuclei in all tissue compartments, as compared with mares in met/dioestrus. For OVX and OVX+Oe mares, there appeared to be a stronger ERα staining intensity and/or higher proportion of stained cells compared with mares in met/dioestrus. No obvious differences in staining intensity or proportion were observed between OVX and OVX+Oe mares. No positive staining was found in the negative controls.

**Table 2:** Immunostaining for ERα in uterine tissue.

Mare ID	Reproductive status	SE	GE	STR	MYO
526	Oestrus	++/+++ D	+++ D	++/+++ D	++/+++ D
478	Metoestrus	-/+ A	-/+ A	+ A	++ D
453	Dioestrus	+/++ B*	+/++ D	+ A	+ A
502	Dioestrus	+ B	+ B	+ A	+ B
740	OVX	++ D	+++ D	++ D	++ D
744	OVX	+ C	+/++ D	++ B	++ D
741	OVX+Oe	+/++ C	++ D	++ C	+ C
742	OVX+Oe	+/++ C	+/++ D	++ C	+ A
743	OVX+Oe	+/++ B	+/++ D	+/++ B	+ D

* Patchy in some areas; OVX: ovariectomised; OVX+Oe: ovariectomised with oestradiol benzoate treatment; SE: surface epithelium; GE: glandular epithelium; STR: connective tissue stroma; MYO: myometrium; A: low proportion (< 30%); B: moderate proportion (30–60%); C: high proportion (> 60–90%); D: almost all cells positive (> 90%); -: negative; +: weak intensity; ++: moderate intensity; +++: strong intensity.

#### Adrenal gland

**Figure 2:**
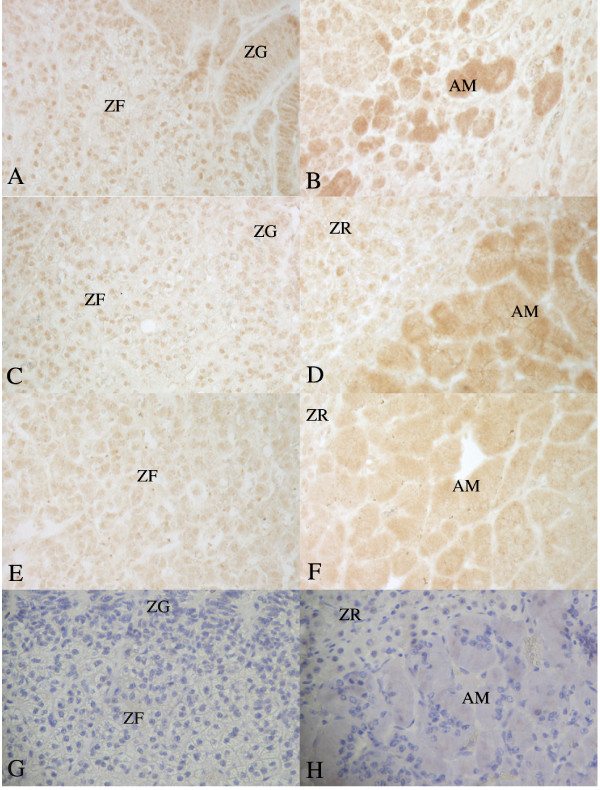
**Immunostaining for ERα and PR in adrenal tissue**. A and B: ERα in mare 744 (OVX); C and D: ERα in mare 502 (intact in dioestrus); E and F: PR in mare 744 (OVX); G and H: negative control. ZG = Zona glomerulosa; ZF = Zona fasciculata; ZR = Zona reticularis and AM = adrenal medulla. Magnification 200×.

For selected results on ERα immunostaining in the adrenal gland, see Fig. 2. In the OVX and OVX+Oe mares, there was a major proportion (>50%) of cell nuclei with moderate ERα staining in all zones of the adrenal cortex. In addition, in the OVX mares, cytoplasmic ERα staining of moderate intensity was also observed in a major proportion of cells in the adrenal medulla. In the cell nuclei of the adrenal cortex of intact mares, the ERα staining intensity was weak, but observed in a major proportion of cells. No specific ERα staining was found in the adrenal medulla of intact mares, except for mare 502, where a minor proportion (<50%) of weak to moderate cytoplasmic ERα staining was observed.

### Immunohistochemistry-progesterone receptor (PR)

#### Uterine tissue

In uterine tissue, positive PR staining was found in the nuclei of all types of uterine cells as for ERα immunostaining (see Table 3 and Fig. 1). The lowest PR intensity and proportion was found in mare 478, a mare considered to be in metoestrus. For the other mares [OVX, OVX+Oe and intact mares (oestrus and dioestrus)], no clear differences were observed. No PR staining was found in the negative controls.

**Table 3:** Immunostaining for PR in uterine tissue.

Mare ID	Reproductive status	SE	GE	STR	MYO
526	Oestrus	++/+++ D	++/+++ D	++/+++ D	+++ D
478	Metoestrus	+ B	+ B	+/++ B	++ D
453	Dioestrus	+++ D	+++ D	+++ D	+++ D
502	Dioestrus	++ D	++/+++ D	++/+++ D	++/+++ D
740	OVX	+/++ D	++ D	+/++ D	+/++ C
744	OVX	+++ D	+++ D	++/+++ D	+++ D
741	OVX+Oe	+/++ D	++ D	++ D	+ D
742	OVX+Oe	++ D	+++ D	+++ D	+/++ D
743	OVX+Oe	++ D	+++ D	+++ D	++ D

OVX: ovariectomised; OVX+Oe: ovariectomised with oestradiol benzoate treatment; SE: surface epithelium; GE: glandular epithelium; STR: connective tissue stroma; MYO: myometrium; A: low proportion (< 30%); B: moderate proportion (30–60%);

C: high proportion (> 60–90%); D: almost all cells positive (> 90%); +: weak intensity; ++: moderate intensity; +++: strong intensity.

### Adrenal gland

For selected results on PR immunostaining in the adrenal gland, see Fig. 2. For PR in all mares, most of the adrenal cortex cells were not stained, but a minor proportion (<50%) of weak positive cells was found in the zona fasciculata and zona reticularis. Moreover, in the cells of the adrenal medulla, a major proportion (>50%) of weak cytoplasmic PR staining was observed in all mare groups.

## Discussion

The positive ERα and PR staining observed in uterine tissue in the present study supports that the monoclonal antibodies that were used correctly identified the receptor proteins. Although the present study did not attempt to investigate the effect of oestrous cycle stage on receptor staining, it was noted that the mare in oestrus showed the strongest staining intensity and highest proportion of stained cells for ERα in all of the uterine compartments studied. This is in accordance with other studies in the mare [16-18], ewe [14], mouse [19] and sow [15] that have showed that ERs are, in general, up-regulated by oestrogens. In studies performed in mares, strong staining for ER was found in cell nuclei of the endometrial connective tissue stroma prior to ovulation [16,18], with either weak [20] or strong [16] nuclear staining for ER in luminal and glandular epithelia during that same period.

In the present study, PR staining in uterine tissue was, in general, found in almost all cells, with a moderate staining intensity. This result on proportion is similar to a study on sow endometrium [21]. However, in the study of Sukjumlong et al. [21], a greater intensity of staining was observed in uterine tissue from sows in oestrus or early dioestrus, indicating an up-regulation of PR staining by oestradiol. Similarly, Hartt et al. [16] found that there appeared to be an up-regulating effect of oestradiol on the level of PR staining in all cell types of the mare endometrium (luminal epithelia, glandular epithelia and stroma), with the highest levels observed during oestrus, close to ovulation. In the present study, the lowest proportion of PR staining was found in mare 478, a mare in metoestrus, which appears contradictory to a stimulatory effect of oestradiol. The reason for the discrepancy between the present findings and the results from other studies is not clear.

In the adrenal gland, ERα staining was found in all three cortical zones and in all mare groups (OVX, OVX+Oe and intact). However, adrenal glands from intact mares showed a lower intensity of ERα staining (weak) compared with OVX and OVX+Oe mares. The results of the present study agree with studies performed in other species. For example, ER staining was found in all adrenal cortex zones in both rhesus monkey [5] and sheep (ER α) [6]. In humans, ERα staining was found only in the zona fasciculata; however, staining for ERβ was present in all three zones [22]. In the present study, OVX mares showed stronger ERα staining in the adrenal cortex compared with intact mares. Similarly, in sheep, long-term gonadectomy (5.5 months) resulted in increased adrenal ER levels in both sexes, as quantified in a LBA [6]. However, in the same study, IHC revealed no effect of gonadectomy on ERα staining intensity in the zona fasciculata. Nonetheless, they speculated that there is an inverse relationship between plasma oestradiol concentrations and adrenal ER levels. In the OVX mares in the present study, oestradiol treatment had no obvious effect on the amount of staining observed (intensity and proportion). The plasma levels of oestradiol in the OVX+Oe mares were similar to those found in mares before ovulation [23], i.e. physiological. In mice and humans, uterine ER levels increased in response to physiological oestradiol levels [20,24]. However, the oestradiol treatment in the present study was most likely of too short duration (18–22 h prior to euthanasia) to have an affect on adrenal ERα staining intensity. The time for euthanasia was chosen due to the well-known rapid effect of oestradiol on oestrous behaviour in the mare. In the ovariectomised ewe, enhancement of ER mRNA and protein expression in most uterine cells required a time of at least 24 h and 48 h, respectively, post-treatment [25]. In addition, in contrast to ovariectomy of longer duration, short-term ovariectomy (1–10 days) in the rat resulted in an initial decrease in adrenal ER binding sites followed by a gradual rise, as assessed by a LBA, indicating several days may be needed for changes in plasma sex steroid concentrations to have an effect on adrenal receptor levels [26]. Similarly, Sukjumlong et al. [15] found the strongest ERα staining in the surface epithelium of the sow uterus during early dioestrus, which may have been due to a delayed effect from the elevated plasma 17-β-oestradiol concentrations at oestrus. The short time-period for oestradiol treatment was, as stated earlier, chosen due to the rapid effect of oestradiol on mare oestrous behaviour. The study would need to be repeated using treatment of a much longer duration in order to draw any conclusions regarding oestratiol treatment and steroid receptor expression in the mare adrenal gland.

In the present study, OVX mares also showed cytoplasmic staining of moderate intensity for ERα in the adrenal medullar cells. ERα staining was also found in the adrenal medulla of sheep [6] and humans [22], but was localised within the cell nuclei. However, little or no medullary ER immunostaining was observed in rhesus monkey [5] and, in a LBA, insignificant [^3^H] oestradiol labelling was observed in the rat adrenal medulla [4]. Indirect evidence indicate that classical ERs may be present in bovine adrenal medullary cells, since the classical ER antagonist ICI182780 blocked the stimulatory effect of 17-β-oestradiol on catecholamine synthesis [27].

The staining in the adrenal medulla observed in the present study was located within the cytoplasm and not the nucleus as in the adrenal cortex. Since ERs are continuously shuttled between the cytoplasm and cell nucleus, some cytoplasmic staining might be expected [28]. However, the marked contrast between cortical and medullary staining (nuclear versus cytoplasmic) was unexpected. In humans, ERα staining was observed in both the cell nuclei and cytoplasm of endometrial luminal epithelial cells [22]. The authors suggested that both nuclear and cytoplasmic ERs are produced by some tissue cells. Furthermore, there is evidence of oestrogen binding receptors in the plasma membrane of bovine adrenal medullary cells. These membrane receptors were seemingly distinct from classical nuclear ERα and ERβ [29]. It has been suggested that oestrogen exerts its effect through adrenal medullary ERs in a rapid, non-genomic manner and therefore most likely through membrane receptors [27,30]. In the present study, equine adrenal medullary cells seem to express cytoplasmic ERα. However, with the method used, we cannot determine if there may also exist membrane bound ERα. Nevertheless, the presence of ERα staining in the equine adrenal medulla may indicate that there could be an effect of oestradiol upon catecholamine secretion in this species. In *in vitro *studies, oestradiol has been shown to affect catecholamine secretion. For example, pharmacological oestradiol doses (1–300 μM) caused an inhibition of catecholamine secretion in PC12 cells (a clonal cell line derived from a transplantable rat adrenal pheochromocytoma) [30], whereas lower doses (0.3–100 nM) stimulated catecholamine secretion in bovine adrenal medullary cells [29]. It is important to note, however, that medullary ER staining in the present study was found predominantly in OVX mares (and only in one intact mare), questioning whether such ER would have any biological effect.

PR was observed in zona fasciculata and reticularis in all mare groups in the present study, although the staining was always weak and occasional. As stated in the introduction, there is conflicting evidence as to the existence of adrenal PR and species differences are apparent. Progesterone binding activity has been demonstrated in nuclei purified from the adrenal cortex of guinea pigs, but the binding protein was considered distinct from classical PR, partly since a monoclonal antibody known to recognise guinea pig classical nuclear uterine PR failed to identify the protein [10]. In the zona fasciculata and zona reticularis of the rat adrenal gland, a protein has been identified as a membrane PR [31]. However, adrenocortical nuclei from several species, including the rat, dog, pig and chinchilla, were found to have no progesterone-binding activity [10]. Similarly, Hirst et al. [5] found no detectable PR staining in adult and fetal adrenal glands from rhesus monkey using IHC.

In the current study, weak PR staining was also observed in the cytoplasm of a major proportion of adrenal medullary cells. To our knowledge, there are no reports on PR in the adrenal medulla in other species. Progesterone has been shown to inhibit catecholamine secretion in bovine chromaffin cells, although this inhibition was attributed to an effect on nicotinic acetylcholine receptors and voltage-dependent calcium channels, and not PR [32]. Further, progesterone and oestradiol were demonstrated to alter catecholamine metabolism in the adrenal medulla of the rat [33]. Thus, progesterone does exert an effect on adrenal medullary function in some species studied and, in view of the present result, this effect may in part involve specific medullary PR. Further studies are required, however, since the present study could only demonstrate weak staining for PR. I

## Conclusion

The present study demonstrated the presence of both ERα and PR immunostaining in the cortex of the mare adrenal gland, although for PR, only weak staining were observed in a minor proportion of cells. To our knowledge, this is the first time ERα and PR in equine adrenal tissue have been investigated. Ovariectomy resulted in stronger cortical ERα immunostaining. The presence of PR and ERα staining in the cytoplasm of adrenal medullary cells was unexpected. Again, ovariectomy influenced the amount of ERα observed, with only one intact mare demonstrating ER staining in the medulla. It is unclear if the steroid receptors found in the mare adrenal gland have any biological effect, and, in particular for PR, further studies are clearly need to verify the presence of this receptor in equine adrenal tissue.

## Abbreviations

ACTH: adrenocorticotrophic hormone; ERα: oestrogen receptor alpha; ERβ: oestrogen receptor beta; HPA: hypothalamo-pituitary adrenal axis; HPG: hypothalamo-pituitary gonadal axis; IHC: immunohistochemistry; i.m.: intramuscularly; LBA: ligand binding assay; mRNA: messenger ribonucleic acid; OVX: ovariectomised; OVX + Oe: oestradiol treated and ovariectomised; PR: progesterone receptor.

## Competing interests

The authors declare that they have no competing interests.

## Authors' contributions

AMD; HK and YHA conceived of the study, participated in its design and collected the adrenal gland and uterine tissues (including weighing and judging reproductive status). SS and YHA performed the immunohistochemistry procedures. SS judged the staining intensities for PR and ER in adrenal and uterine tissue. YHA carried out the hormone analyses and drafted the manuscript. All authors read and approved the final manuscript.
